# Deep Neural Network-Based Automatic Dicentric Chromosome Detection Using a Model Pretrained on Common Objects

**DOI:** 10.3390/diagnostics13203191

**Published:** 2023-10-12

**Authors:** Kangsan Kim, Kwang Seok Kim, Won Il Jang, Seongjae Jang, Gil Tae Hwang, Sang-Keun Woo

**Affiliations:** 1 Division of Applied RI, Korea Institute of Radiological and Medical Sciences, Seoul 01812, Republic of Korea; krmount@kirams.re.kr; 2 Department of Radiation Oncology, Korea Institute of Radiological and Medical Sciences, Seoul 01812, Republic of Korea; 3 National Radiation Emergency Medical Center, Korea Institute of Radiological and Medical Sciences, Seoul 01812, Republic of Korea; sjsjj@kirams.re.kr; 4 Department of Chemistry and Green-Nano Materials Research Center, Kyungpook National University, Daegu 41566, Republic of Korea; giltae@knu.ac.kr

**Keywords:** dicentric chromosome assay, cytogenetic dosimetry, chromosome metaphases image, object detection, you only look once, deep learning, transfer learning

## Abstract

Dicentric chromosome assay (DCA) is one of the cytogenetic dosimetry methods where the absorbed dose is estimated by counting the number of dicentric chromosomes, which is a major radiation-induced change in DNA. However, DCA is a time-consuming task and requires technical expertise. In this study, a neural network was applied for automating the DCA. We used YOLOv5, a one-stage detection algorithm, to mitigate these limitations by automating the estimation of the number of dicentric chromosomes in chromosome metaphase images. YOLOv5 was pretrained on common object datasets. For training, 887 augmented chromosome images were used. We evaluated the model using validation and test datasets with 380 and 300 images, respectively. With pretrained parameters, the trained model detected chromosomes in the images with a maximum F1 score of 0.94 and a mean average precision (mAP) of 0.961. Conversely, when the model was randomly initialized, the training performance decreased, with a maximum F1 score and mAP of 0.82 and 0.873%, respectively. These results confirm that the model could effectively detect dicentric chromosomes in an image. Consequently, automatic DCA is expected to be conducted based on deep learning for object detection, requiring a relatively small amount of chromosome data for training using the pretrained network.

## 1. Introduction

Biological dosimetry is a retrospective method for estimating the absorbed radiation dose of patients based on biological endpoints. Among the various methods in biological dosimetry, cytogenetic dosimetry is the most widely used, especially for the early triage of radiation mass casualties [1]. Cytogenetic dosimetry utilizes the nature of the radiation–DNA interaction to estimate absorbed doses. Ionizing radiation induces DNA damage in two ways: directly by ionizing the DNA molecule or indirectly by generating free radicals [2,3,4]. Chromosome aberrations, including deletion, duplication, inversion, and translocation, are likely to occur when the damaged DNA, especially double-strand breaks, is repaired by the cellular DNA repair system [5,6,7]. The frequency of chromosome aberrations can be estimated by counting them while acquiring Giemsa-stained images of the metaphase stage. Subsequently, the absorbed dose is estimated by the counts based on the linear-quadratic model between the frequency of chromosome aberration and the absorbed dose according to the radiation type. Cytogenetic dosimetry involves several methods, such as the dicentric chromosome assay (DCA), cytokinesis-blocked micronucleus assay, translocation assay, and premature chromosome condensation assay [1]. 

DCA is one of the most widely used cytogenetic dosimetry methods that measures the frequency of a dicentric chromosome, which is an abnormal chromosome with two centromeres [8,9]. It is created owing to the misrepair of two chromosomes and abnormal chromosome replication, and the most commonly occurring abnormal chromosome is generated by irradiation. The background frequency of the dicentric chromosome in the normal population is approximately 1 per 1000 cells. A major characteristic of the dicentric chromosome is that it is extremely sensitive to radiation to the extent that the threshold dose is just approximately 0.1 Gy. These characteristics make DCA the gold standard for biological dosimetry. However, DCA is a highly expertise-dependent and time-consuming task when it comes to its application in the early triage of mass casualties. 

Studies have been conducted to automate DCA using machine learning [10,11] and deep learning [12,13,14] by using it to construct dose–response curves and calculate estimated doses [13] or as a classifier for individual chromosome patches extracted from images of individual metaphases [14]. In addition, several studies have been conducted on chromosome classification based on deep learning [15,16], and there have been studies on deep learning-based segmentation for biomedical images [17,18,19]. However, applying those findings to automate DCA has critical limitations because additional segmentation or localization methods are required before classification to obtain individual chromosome data within the image. Therefore, we consider that an object detection method can provide the appropriate framework for automated DCA, which outputs the number of dicentric chromosomes from a chromosome metaphase image.

Object detection is a computer vision task that aims to identify the objects within an image or video and classify them [20,21,22]. The rapid progress in deep learning in the field of computer vision in recent decades has resulted in the advancement of object detection techniques by adopting a convolution neural network [23,24,25] or the vision transformer [26,27,28] as their feature extraction backbone. Most of the object detection models aim to find the position of instances in an image and classify them from extracted image features. Considering their focus on finding and classifying instances within an image, object detection methods have potential applications in automating DCA.

**Figure 1:**
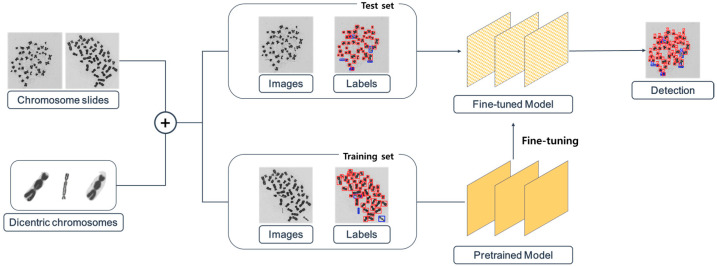
Comprehensive procedure for training the object detection network with chromosome images and individual dicentric chromosome patches. The object detection model is initialized with the pretrained weight, regardless of the chromosome or the metaphase image.

This study introduces the “You Only Look Once” (YOLO) algorithm [25], which is a widely used deep learning-based object detection algorithm, to the automation of DCA. Because the object detection model aims to classify the objects within the image, the model was directly applied to perform both object localization within the chromosome metaphase image and their classification. We attempted to enhance model performance by using pretrained parameters and treating the problem as a downstream task. In addition, since the ratio of the monocentric and dicentric chromosomes is imbalanced, we applied the augmentation technique to address the class imbalance issue. The overall pipeline is illustrated in Figure 1.

## 2. Materials and Methods

YOLO is a one-stage object detection algorithm that localizes the bounding box and simultaneously classifies it [25,29,30]. In YOLO, a global image is used as the network input, and the detection result is the output, which is a single vector integrating the positional and geometric information of the bounding box and its classification. More specifically, the output of YOLO is the concatenation of the four positional elements, which determine the position of the bounding box in the image; the confidence score, which is the probability of whether the object belongs to the bounding box or not; and the class probabilities for classes. Thus, the object detection task is converted into a single regression problem in the YOLO architecture. Owing to its simplicity, YOLO is fast, and this is a major advantage when adopting the method for DCA automation. Several subsequent versions of YOLO have been published and have improved detection performance [31]. In this study, YOLOv5 [32], a recent version of the YOLO family, was used as the object detection model.

In the YOLO framework, the input image is divided into several grids. Each grid cell is supposed to contain one class. The number of predicted bounding boxes for the input image is proportional to the number of grid cells. The positional information of the bounding box in the network output is related to its relative position on the grid. Moreover, several anchor boxes are assigned to each grid. The dimensions of the anchor boxes are initially determined using K-means clustering to choose the bounding boxes from the training set and the intersection over union (IoU)-based distance. The size of the bounding box is determined by adjusting the size of the anchor box. Therefore, the network predicts as many bounding boxes as the number of grids × number of anchor boxes when a single image is inputted.

One of the problems with most object detection models is that they create multiple bounding boxes for an object. The non-maximum suppression (NMS) [33] method is adopted to select the most significant bounding box. In NMS, the bounding boxes for which IoU with the bounding box of the highest confidence score is higher than the threshold are filtered out.

**Figure 2:**
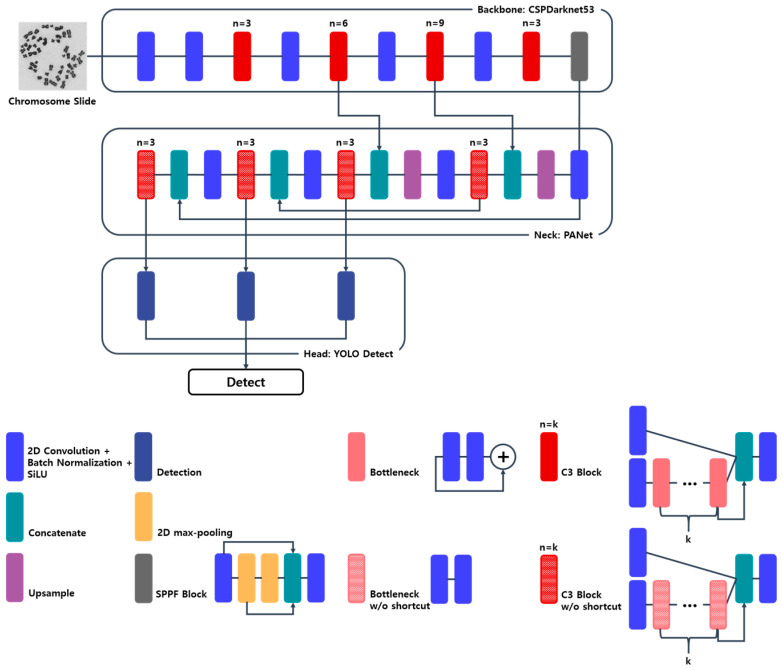
Network architecture of YOLOv5. The network consists of a backbone (CSPDarknet), neck (PANet), and head (YOLO layer).

The network structure used in the recent versions of YOLO consists of three parts: the backbone, neck, and head. Since an image is used as the network input, each part is constructed based on a 2-dimensional convolution neural network. In YOLOv3, multi-scale features extracted in the backbone are used for object detection. The detailed network structure is illustrated in Figure 2. The backbone extracts image features. In YOLOv5, CSPDarknet, a modified version of Darknet that uses a cross-stage partial network (CSPNet) [34] in its residual blocks, is used as the backbone. The path aggregation network (PANet) [35] is used as the neck. PANet is based on the feature pyramid network (FPN) [36], which prevents features from the backbone’s lower stage from being ignored. Finally, the features modified by the neck proceed to the head, which converts them into the output, which includes localization and classification information. YOLOv5 has various model sizes. YOLOv5s, the second-smallest and fastest YOLOv5 network model, was used in our study, and it has 7.2 million parameters to be trained.

The loss functions of YOLOv5 consist of three parts: location loss function, classes loss function, and objectness loss function. These mainly originate from the tasks the one-stage object detection algorithm is designed to perform: localize the bounding boxes, verify whether the object is in the box, and classify the object. Location loss function is related to the bounding box geometry and its location. Among the elements in the output vector, each bounding box’s four-dimensional information (t_x_, t_y_, t_w_, t_h_) is transformed into the bounding box geometry as Equation (1). (*C_x_*, *C_y_*) is the coordinate of the input grid.

(1) $$b_{x}=sigmoid\left(t_{x}\right)+C_{x}\;b_{y}=sigmoid\left(t_{y}\right)+C_{y}\;b_{w}=p_{w}\exp\left(t_{w}\right)\;b_{h}=p_{h}\exp\left(t_{h}\right)$$

The sigmoid function is defined as $\;sigmoid\left(x\right)={\left(1+e^{-x}\right)}^{-1}$. Location loss function is calculated based on the IoU of the bounding box from the network output and its ground truth. In YOLOv5, the complete IoU (CIoU) [37] is used as a location loss function. It considers the IoU of the ground-truth bounding box, the generated bounding box, the distance between their centers, and the aspect ratio. For the diagonal length *c* of the enclosing box of prediction box *b*, its ground truth *b^gt^*, and the distance between their center *ρ*(*b*,*b^gt^*), the CIoU is calculated using Equation (2).

(2) $$L_{loc}=1-IoU+\frac{\rho^{2}\left(b,b^{gt}\right)}{c^{2}}+\alpha \upsilon \;\upsilon =\frac{4}{\pi^{2}}{\left(\arctan\frac{b_{w}^{gt}}{b_{h}^{gt}}-\arctan\frac{b_{w}}{b_{h}}\right)}^{2}\;\alpha =\frac{\upsilon }{\left(1-IoU\right)+\upsilon }$$

Objectness loss function is formulated based on the binary cross entropy of the confidence score of the bounding box. The bounding boxes generated from the network generally do not contain the object when calculating the objectness loss function, and the class imbalance problem [38] can occur. Hence, the objectness loss function is calculated as the weighted sum of the binary cross entropy of the bounding boxes from the output, with much less weight attached to the bounding box with no object. The formulation is expressed as

(3) $$L_{obj}=-\sum_{i}^{S^{2}}\sum_{j}^{B}I_{ij}^{obj}CE\left(C_{i},\hat{C_{i}}\right)-\lambda_{noobj}\sum_{i}^{S^{2}}\sum_{j}^{B}I_{ij}^{noobj}CE\left(C_{i},\hat{C_{i}}\right).$$

The loss function of classes is calculated as the sum of the binary cross entropy of the class probability but only for the bounding boxes that include the object. The entire formulation is expressed as

(4) $$L_{class}=-\sum_{i}^{S^{2}}I_{ij}^{obj}\sum_{c\in classes}CE\left(p_{i}\left(c\right),{\hat{p}}_{i}\left(c\right)\right).$$

Metaphase images with or without chromosomal aberrations were provided by the National Radiation Emergency Medical Center at the Korea Institute of Radiological and Medical Sciences [39,40]. A total of 1456 Giemsa-staining metaphase images were gathered, and 189 of them included a dicentric chromosome.

The position of the bounding boxes, which include a normal chromosome in the image, was acquired by using Otsu’s algorithm and the “regionprops” method in the scikit-learn library in Python for labeling. Conversely, the boxes bounding the dicentric chromosome were selected manually. As there were many more normal chromosomes than dicentric ones in the image, the class imbalance problem had to be mitigated. Therefore, individual dicentric chromosome patches were added to the image to mitigate the problem while avoiding superposition over the chromosomes. By segmenting the chromosomes using Otsu’s algorithm, the backgrounds in the patches were made transparent before addition. Moreover, several normal individual chromosome patches were similarly attached to prevent the model from being trained to detect the dicentric chromosome by its segmented edge. The number of dicentric chromosome bounding boxes was 2133 in the training dataset, while it was 143 before augmentation.

We split the dataset into a training set; a validation dataset, which included the augmented dicentric chromosome patches; and a test dataset, which included dicentric chromosomes and did not have an augmented slide. The numbers of chromosome metaphase images within those datasets were 887, 380, and 189, respectively. Originally, the images in the dataset differed in size; however, they were resized to 640 × 640 pixels before being used as the input for the network.

The number of epochs was set to 200, and the size of the mini-batch was 32. The model was optimized using the stochastic gradient descent method. When training the network without any information, we used a fine-tuning technique to enhance the model’s performance. The network parameters were initialized with pretrained weights, which were trained using the Microsoft Common Objects in Context (MS-COCO) dataset [41], which is unrelated to chromosome images. 

The performance of the object detection model was mainly evaluated using confusion matrix-based metrics, such as precision and recall. Precision is defined as the ratio of the number of true positive samples to the number of positive samples labeled by the prediction model. On the other hand, recall is defined as the ratio of the number of true positive samples to the real number of positive samples. These metrics are affected by IoU thresholds and a confidence threshold, which determine whether the object is in the bounding box or classified as a specific class and whether the proposed bounding box coincides with the ground truth. Therefore, this study evaluated model performance by setting the precision and recall above the confidence threshold, while the IoU threshold was fixed at 0.5. The F1 score and average precision (AP), typically employed in object detection, were used as the evaluation metrics. The F1 score is defined as the harmonic mean of precision and recall, and the area under the precision–recall curve calculates the AP. The mean AP (mAP) is the mean of the AP over the classification category.

## 3. Results

### 3.1. Convergence in Training

**Figure 3:**
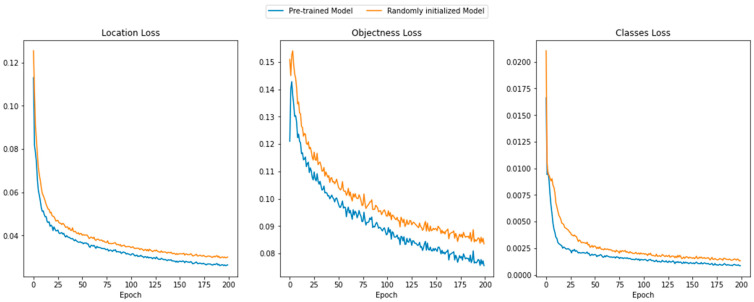
Plots of training losses (location loss, objectness loss, and classes loss) versus the epochs for the pretrained and randomized initial weights.

Figure 3 plots the behavior of the loss functions according to the epochs during training for both the pretrained and randomly initialized models. The loss functions decreased with the epochs, implying that the training process was sufficiently stable for both cases. Moreover, the losses of the pretrained model were always lower than those of the randomly initialized one for the entire training process; however, it was not capable of detecting or distinguishing the individual chromosomes before training. These findings show that the detecting capability of the model trained with an object unrelated to the chromosome image of the Giemsa-stained image was utilized appropriately in the training process.

### 3.2. Evaluation

**Figure 4:**
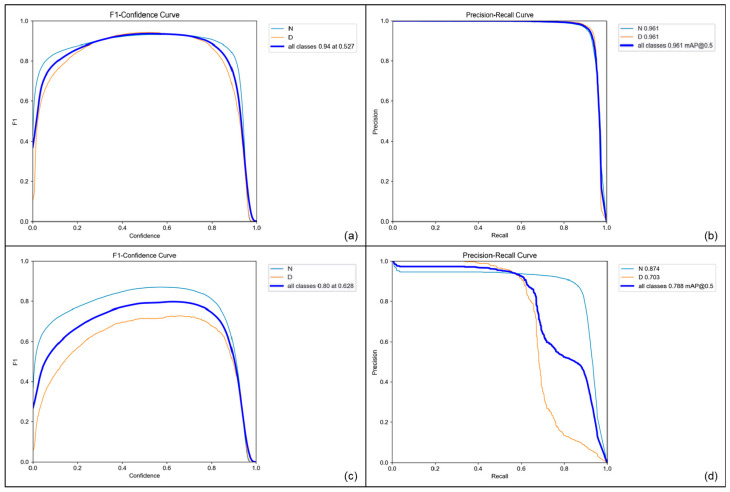
Plots of evaluation metrics for the model pretrained on the MS-COCO dataset. (**a**) F1 score vs. confidence score and (**b**) precision–recall curve for validation dataset. (**c**) F1 score vs. confidence score and (**d**) precision–recall curve for test dataset. “N” and “D” denote normal and dicentric chromosome, respectively.

Figure 4 illustrates the evaluation results of the fine-tuned model on both validation and test sets. In the validation set, both normal and dicentric chromosomes were appropriately detected. The maximum F1 score was approximately 0.94 when the confidence score was 0.527. Moreover, the model accurately detected normal and dicentric chromosomes in terms of mAP, scoring 0.961 for the IoU threshold of 0.5. Specifically, the precision and recall for normal chromosomes were 0.946 and 0.915, respectively, whereas those for the dicentric ones were 0.962 and 0.921, respectively.

The models were also evaluated on the test dataset with chromosome metaphase images without any augmentation. On the test dataset, it is shown that the maximum F1 score was 0.80 when the confidence score was 0.628. In addition, when the IoU threshold was 0.5, the mAPs for normal and dicentric chromosomes were 0.874 and 0.703, respectively. The precision and recall for predicting normal chromosomes were 0.896 and 0.842, while those for dicentric chromosomes were 0.886 and 0.615. 

**Figure 5:**
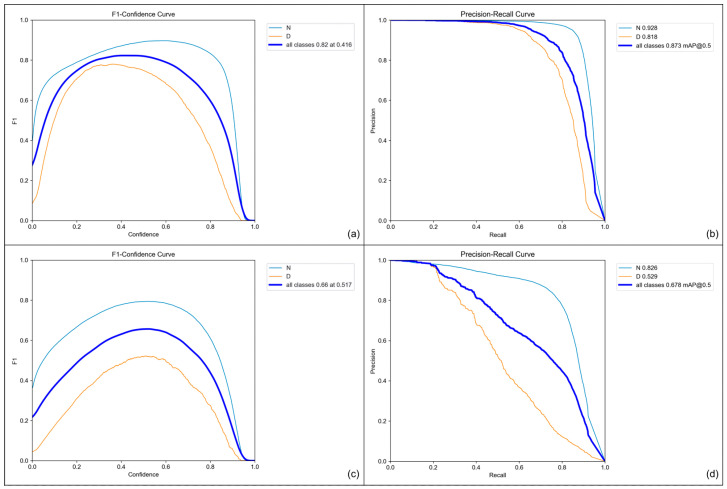
Plots of evaluation metrics for the randomly initialized model. (**a**) F1 score vs. confidence score and (**b**) precision–recall curve for the validation dataset. (**c**) F1 score vs. confidence score and (**d**) precision–recall curve for the test dataset. “N” and “D” denote normal and dicentric chromosome, respectively.

Conversely, the evaluation metrics over the confidence score deteriorated when the model was not pretrained, as shown in Figure 5. As shown in the figure, the maximum F1 score was 0.82 when the confidence score was 0.416 and the mAPs of the normal chromosome, dicentric chromosome, and all classes were 0.928, 0.818, and 0.873, respectively, for the validation dataset. The comparison of the precision–recall curve and F1–confidence curve between the pretrained network and the randomly initialized one demonstrates that pretraining contributed to enhancing the performance of detecting dicentric chromosomes. This tendency becomes more obvious when it comes to evaluating the performance of the test dataset. The F1 score of the randomly initialized network was 0.66 for all classes at a 0.517 confidence score. Moreover, the mAPs of the normal, dicentric, and whole chromosomes were 0.826, 0.529, and 0.678, respectively. While the performance of detecting normal chromosomes slightly degraded, the detection performance for the dicentric ones decreased significantly. 

The overall results for the comparison of using the pretrained network are listed in Table 1. According to the table, using the pretrained weight enhanced the performance of the model. Moreover, based on the results for the test dataset shown in Figure 4 and Figure 5, the model was less effective in detecting dicentric chromosomes than detecting normal ones. However, by adopting the pretrained weight with the MS-COCO dataset, the performance improved significantly in terms of detecting dicentric chromosomes, while the performance achieved in detecting normal ones was relatively insensitive to the initial weight of the object detection model.

**Figure 6:**
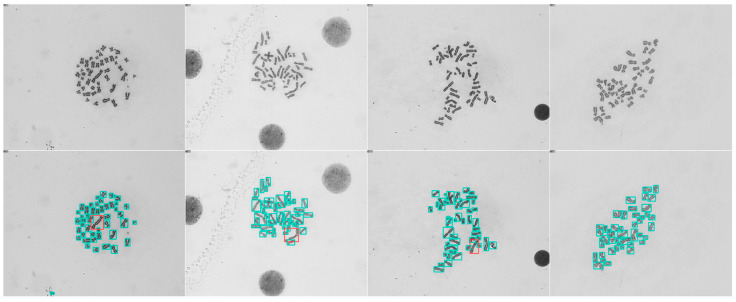
Visualization examples of the input (first row) and corresponding detection results of the input (second row). The red bounding boxes contain the dicentric chromosomes and the green ones the normal monocentric chromosomes.

Figure 6 visually compares the ground-truth label with the object detection model output. Furthermore, we evaluated the mean number of chromosomes per metaphase to evaluate how close they came to 46 chromosomes per metaphase. While the number of chromosomes per metaphase in the validation set was 45.92, the predicted number of chromosomes, including both normal and dicentric ones, per metaphase was 47.32.

## 4. Discussion

Our experiments showed that normal and dicentric individual chromosome detection is more accurate when using a weight pretrained on the MS-COCO dataset consisting of unrelated common objects. Specifically, the capability to classify both normal and dicentric chromosomes becomes considerably better when using the pretrained network. The network used in the object detection algorithm has numerous parameters to be trained; thus, it is obvious that enormous amounts of data should be prepared for training. However, since both collecting and labeling the chromosome metaphase images are complex and expertise-dependent tasks, acquiring an adequately sized dataset is hard and costly. In this point of view, our results indicate that pretraining can improve model performance and address the challenge of creating and labeling a large dataset, which is time-consuming and requires expertise.

As shown in Figure 3, the models were trained stably for both cases, representing similar values for three kinds of losses. However, the evaluation results were quite different from each other, and, even for the test dataset, the randomly initialized model shows very poor performance in detecting dicentric chromosomes. This originates from the overfitting issue of the model, since the dataset is not large enough for training. Therefore, it is directly related to the advantage of using a pretrained network, which mitigates the issue. Moreover, the recall value on the test dataset shows that the model tends to predict relatively high false negatives for dicentric chromosomes, which originates from the class imbalance problem in both classes. The main issue in the automation of DCA using deep learning is the nature in which the dicentric chromosomes are underrepresented in the chromosome metaphase image. Although individual dicentric chromosome patches were added to the chromosome images to mitigate the class imbalance problem, they did not essentially solve the problem. Moreover, acquiring numerous chromosome images that include an adequate number of dicentric chromosomes is crucial. However, labeling a sizeable dicentric chromosome dataset, especially for DCA, would be expensive owing to the need for specialized expertise. Therefore, a semi-supervised learning model [42,43] should be developed to deal with a significant, partially labeled dataset. Recently, numerous studies on semi-supervised object detection have been conducted [44,45]. To practically implement automated DCA using an object detection model, a suitable semi-supervised architecture must be used and validated. 

Studies have been conducted to automate DCA by adopting a deep learning model. Jang et al. [12] suggested a deep learning-based automated dose estimation system (DLADES) for DCA automation and absorbed dose estimation. They used faster R-CNN, a deep learning object detection algorithm with FPN. The automation network was composed of the counting network (CN) and identifying network (IN). Trained on 3031 images, the precision and recall of the CN were 97.8% and 97.9%, respectively, and those for IN, trained with 9904 images, were 90.5% and 90.4%, respectively. Wadhwa et al. [14] minimized the intervention of field experts by introducing an objection detection model only to extract the chromosome patches regardless of their abnormality. Subsequently, the dicentric classifier was applied to the extracted individual chromosome patches. Trained on 4.5–5 Gy images from WHO-BIODOSENET, the model achieved 98.54% and 90% precision and recall, respectively, when using the Inception Resnet V2 network as the dicentric classifier. Compared to the performance shown in the results section, our model, where the object detection algorithm is directly applied, was less effective in terms of mAP, scoring 0.8, than the others. However, our approach using the pretraining is compatible with the other method, so it can be utilized even for those methods. 

There are some limitations of this study. Because the internal test dataset cannot evaluate the overfitting of the model properly, an external test set acquired by another institute or protocol is required for the evaluation of the trained model. Therefore, we will evaluate the model with an external dataset in the future. In addition, the dose–response curve should be estimated from the predicted number of dicentric chromosomes. As the relationship between the estimated dose and dicentric chromosome frequency can be expressed as a linear-quadratic function, the dose–response curve can be obtained by fitting the following model:

(5) $$F=\alpha D^{2}+\beta D+\gamma$$

where *F* is the frequency of the dicentric chromosome; D is the absorbed dose; and α, β, and γ are the parameters to be fitted. The linear-quadratic curve can be fitted to the training data for which the amount of irradiated dose is given. After fitting, the absorbed dose can be estimated by solving the quadratic equation with the estimated dicentric chromosome frequency using deep learning. We expect that sequential application of the object detection deep learning model and linear-quadratic dose–response curve can function as an end-to-end automatic cytogenetic dosimetry tool that outputs the estimated absorbed dose from the set of the chromosome metaphase images. Consequently, it is expected that the deep learning-based dicentric chromosome assay will mitigate the expertise-dependent and time-consuming limitations of DCA simultaneously.

## 5. Conclusions

DCA is a cytogenetic dosimetry method for measuring radiation-induced DNA damage. Counting dicentric chromosome by hand is time-consuming and requires expertise. In this study, YOLOv5 was applied to chromosome images to examine the applicability of methods for automating DCA. It was remarkable that using images from the MS-COCO dataset that were unrelated to the target chromosome images to pretrain the weights clearly improved the performance of the detection model. The strategy also has significant advantages in preparing the appropriate number of data for training, since it requires a high cost for the chromosome metaphase image and its labeling. In addition, individual dicentric chromosome patches were used to alleviate the class imbalance problem. However, the training dataset with dicentric ones could be expanded. In practice, the labeling cost must be resolved to obtain a large dataset and should precede research on a semi-supervised object detection model for DCA. Moreover, the dose is expected to be estimated automatically using the automatic DCA deep learning model with the linear-quadratic dose–response curve together, where both are trained and fitted with the same dataset and the amount of the absorbed dose of the subjects is given. It is expected that applying the object detection deep learning model with a pretrained weight that is trained with the dataset with chromosome-regardless objects can be used in real applications or other studies, if further studies that resolve those limitations are conducted.

## Figures and Tables

**Table 1:** Comparison of model performance according to the initialization of the network.

Initialization	Dataset	F1 Score	mAP
Pretrained	Validation	0.94	0.961
	Test	0.80	0.788
Random	Validation	0.82	0.873
	Test	0.66	0.678

## Data Availability

The data that support the findings of this study are available from the corresponding author upon reasonable request.

## References

[1] Gnanasekaran T.S. (2021). Cytogenetic Biological Dosimetry Assays: Recent Developments and Updates. Radiat. Oncol. J..

[2] Téoule R. (1987). Radiation-Induced DNA Damage and Its Repair. Int. J. Radiat. Biol. Relat. Stud. Phys. Chem. Med..

[3] Lomax M.E., Folkes L.K., O’Neill P. (2013). Biological Consequences of Radiation-Induced DNA Damage: Relevance to Radiotherapy. Clin. Oncol..

[4] Hall J., Angèle S. (1999). Radiation, DNA Damage and Cancer. Mol. Med. Today.

[5] Pfeiffer P., Goedecke W., Obe G. (2000). Mechanisms of DNA Double-Strand Break Repair and Their Potential to Induce Chromosomal Aberrations. Mutagenesis.

[6] Iliakis G., Wang H., Perrault A.R., Boecker W., Rosidi B., Windhofer F., Wu W., Guan J., Terzoudi G., Panteliasc G. (2004). Mechanisms of DNA Double Strand Break Repair and Chromosome Aberration Formation. Cytogenet. Genome Res..

[7] Durante M., Bedford J.S., Chen D.J., Conrad S., Cornforth M.N., Natarajan A.T., van Gent D.C., Obe G. (2013). From DNA Damage to Chromosome Aberrations: Joining the Break. Mutat. Res. Toxicol. Environ. Mutagen..

[8] Lloyd D.C., Edwards A.A., Moquet J.E., Guerrero-Carbajal Y.C. (2000). The Role of Cytogenetics in Early Triage of Radiation Casualties. Appl. Radiat. Isot..

[9] Voisin P., Benderitter M., Claraz M., Chambrette V., Sorokine-Durm I., Delbos M., Durand V., Leroy A., Paillole N. (2001). The Cytogenetic Dosimetry of Recent Accidental Overexposure. Cell. Mol. Biol..

[10] Shirley B., Li Y., Knoll J.H.M., Rogan P.K. (2017). Expedited Radiation Biodosimetry by Automated Dicentric Chromosome Identification (ADCI) and Dose Estimation. JoVE J. Vis. Exp..

[11] Shuryak I., Royba E., Repin M., Turner H.C., Garty G., Deoli N., Brenner D.J. (2022). A Machine Learning Method for Improving the Accuracy of Radiation Biodosimetry by Combining Data from the Dicentric Chromosomes and Micronucleus Assays. Sci. Rep..

[12] Jang S., Shin S.G., Lee M.J., Han S., Choi C.H., Kim S., Cho W.S., Kim S.H., Kang Y.R., Jo W. (2021). Feasibility Study on Automatic Interpretation of Radiation Dose Using Deep Learning Technique for Dicentric Chromosome Assay. Radiat. Res..

[13] Jeong S.K., Oh S.J., Kim S.H., Jang S., Kang Y.R., Kim H.J., Kye Y.U., Lee S.H., Lee C.G., Park M.T. (2022). Dicentric Chromosome Assay Using a Deep Learning-Based Automated System. Sci. Rep..

[14] Wadhwa A.S., Tyagi N., Chowdhury P.R. (2022). Deep Learning Based Automatic Detection of Dicentric Chromosome. arXiv.

[15] Zhang W., Song S., Bai T., Zhao Y., Ma F., Su J., Yu L. Chromosome Classification with Convolutional Neural Network Based Deep Learning. Proceedings of the 2018 11th International Congress on Image and Signal Processing, BioMedical Engineering and Informatics (CISP-BMEI).

[16] Wang C., Yu L., Zhu X., Su J., Ma F. (2020). Extended ResNet and Label Feature Vector Based Chromosome Classification. IEEE Access.

[17] Liu Y., Han G., Liu X. (2022). Lightweight Compound Scaling Network for Nasopharyngeal Carcinoma Segmentation from MR Images. Sensors.

[18] Guo S., Liu X., Zhang H., Lin Q., Xu L., Shi C., Gao Z., Guzzo A., Fortino G. (2023). Causal Knowledge Fusion for 3D Cross-Modality Cardiac Image Segmentation. Inf. Fusion.

[19] Zhuang Z., Yang Z., Raj A.N.J., Wei C., Jin P., Zhuang S. (2021). Breast Ultrasound Tumor Image Classification Using Image Decomposition and Fusion Based on Adaptive Multi-Model Spatial Feature Fusion. Comput. Methods Programs Biomed..

[20] Zhao Z.Q., Zheng P., Xu S.T., Wu X. (2019). Object Detection with Deep Learning: A Review. IEEE Trans. Neural Netw. Learn. Syst..

[21] Zou Z., Shi Z., Guo Y., Ye J. (2019). Object Detection in 20 Years: A Survey. arXiv.

[22] Zhiqiang W., Jun L. A Review of Object Detection Based on Convolutional Neural Network. Proceedings of the 2017 36th Chinese Control Conference (CCC).

[23] Ren S., He K., Girshick R., Sun J. Faster R-CNN: Towards Real-Time Object Detection with Region Proposal Networks. Proceedings of the Advances in Neural Information Processing Systems 28 (NIPS 2015).

[24] He K., Gkioxari G., Dollar P., Girshick R. Mask R-CNN. Proceedings of the IEEE International Conference on Computer Vision (ICCV).

[25] Redmon J., Divvala S., Girshick R., Farhadi A. You Only Look Once: Unified, Real-Time Object Detection. Proceedings of the 2016 IEEE Conference on Computer Vision and Pattern Recognition (CVPR).

[26] Beal J., Kim E., Tzeng E., Huk D., Andrew P., Dmitry Z., Pinterest K. (2020). Toward Transformer-Based Object Detection. arXiv.

[27] Zhang Z., Lu X., Cao G., Yang Y., Jiao L., Liu F. ViT-YOLO: Transformer-Based YOLO for Object Detection. Proceedings of the 2021 IEEE/CVF International Conference on Computer Vision (ICCV) Workshops.

[28] Li Y., Mao H., Girshick R., He K. Exploring Plain Vision Transformer Backbones for Object Detection. Proceedings of the Computer Vision—ECCV 2022.

[29] Redmon J., Farhadi A. YOLO9000: Better, Faster, Stronger. Proceedings of the 2017 IEEE Conference on Computer Vision and Pattern Recognition (CVPR).

[30] Redmon J., Farhadi A. (2018). YOLOv3: An Incremental Improvement. arXiv.

[31] Jiang P., Ergu D., Liu F., Cai Y., Ma B. (2022). A Review of Yolo Algorithm Developments. Procedia Comput. Sci..

[32] Jocher G., Stoken A., Chaurasia A., Borovec J., Kwon Y., Michael K., Changyu L., Fang J., NanoCode012, TaoXie (2021). Ultralytics/Yolov5: V6.0—YOLOv5n “Nano” Models, Roboflow Integration, TensorFlow Export, OpenCV DNN Support. Zenodo.

[33] Neubeck A., Van Gool L. (2006). Efficient Non-Maximum Suppression. Proc.—Int. Conf. Pattern Recognit..

[34] Wang C.-Y., Liao H.-Y.M., Wu Y.-H., Chen P.-Y., Hsieh J.-W., Yeh I.-H. CSPNet: A New Backbone That Can Enhance Learning Capability of CNN. Proceedings of the2020 IEEE/CVF Conference on Computer Vision and Pattern Recognition (CVPR) Workshops.

[35] Liu S., Qi L., Qin H., Shi J., Jia J. Path Aggregation Network for Instance Segmentation. Proceedings of the 2018 IEEE Conference on Computer Vision and Pattern Recognition (CVPR).

[36] Lin T.-Y., Dollar P., Girshick R., He K., Hariharan B., Belongie S. Feature Pyramid Networks for Object Detection. Proceedings of the 2017 IEEE Conference on Computer Vision and Pattern Recognition (CVPR).

[37] Zheng Z., Wang P., Liu W., Li J., Ye R., Ren D. (2020). Distance-IoU Loss: Faster and Better Learning for Bounding Box Regression. Proc. AAAI Conf. Artif. Intell..

[38] Oksuz K., Cam B.C., Kalkan S., Akbas E. (2021). Imbalance Problems in Object Detection: A Review. IEEE Trans. Pattern Anal. Mach. Intell..

[39] Lee Y., Jin Y.W., Wilkins R.C., Jang S. (2019). Validation of the Dicentric Chromosome Assay for Radiation Biological Dosimetry in South Korea. J. Radiat. Res..

[40] Lee Y., Seo S., Jin Y.W., Jang S. (2019). Assessment of Working Environment and Personal Dosimeter-Wearing Compliance of Industrial Radiographers Based on Chromosome Aberration Frequencies. J. Radiol. Prot..

[41] Lin T.Y., Maire M., Belongie S., Hays J., Perona P., Ramanan D., Dollár P., Zitnick C.L. Microsoft COCO: Common Objects in Context. Proceedings of the Computer Vision—ECCV 2014.

[42] van Engelen J.E., Hoos H.H. (2020). A Survey on Semi-Supervised Learning. Mach. Learn..

[43] Zhou Z.-H. (2021). Semi-Supervised Learning. Machine Learning.

[44] Liu Y.-C., Ma C.-Y., He Z., Kuo C.-W., Chen K., Zhang P., Wu B., Kira Z., Vajda P. (2021). Unbiased Teacher for Semi-Supervised Object Detection. arXiv.

[45] Tang P., Ramaiah C., Wang Y., Xu R., Xiong C. Proposal Learning for Semi-Supervised Object Detection. Proceedings of the 2021 IEEE Winter Conference on Applications of Computer Vision (WACV).

